# Warfarin Quality Metrics for Hospitalized Older Adults

**DOI:** 10.1055/s-0038-1667138

**Published:** 2018-07-18

**Authors:** Jessica Cohen, Liron Sinvani, Jason J. Wang, Andrzej Kozikowski, Vidhi Patel, Guang Qiu, Renee Pekmezaris, Alex C. Spyropoulos

**Affiliations:** 1 Division of Hospital Medicine, Department of Medicine, Northwell Health, Manhasset, New York, United States; 2 Donald and Barbara Zucker School of Medicine at Hofstra/Northwell, Northwell Health, Manhasset, New York, United States; 3 Division of Health Services Research, Center for Health Innovations and Outcomes Research, Department of Medicine, Northwell Health, Manhasset, New York, United States; 4 Department of Medicine, Anticoagulation and Clinical Thrombosis Services, Northwell Health System, Lenox Hill Hospital, New York, New York, United States

**Keywords:** adverse drug events, anticoagulants, inpatients, INR, warfarin

## Abstract

**Background** Warfarin's adverse drug events are dangerous, common, and costly. While outpatient warfarin management tools exist, there is a dearth of guidance for inpatients.

**Objectives** We sought to describe a health system's chronic warfarin quality metrics in older inpatients, defined by international normalized ratio (INR) control, explore associations between INR overshoots and clinical outcomes, and identify factors associated with overshoots.

**Patients/Methods** Data on patients 65 years and older who were prescribed chronic warfarin and admitted during January 1, 2014, to June 30, 2016, were extracted through retrospective chart review. We defined overshoots as INRs 5 or greater after 48 hours of hospitalization. Logistic regression modeling was used to determine risks for overshoots and multivariate analysis for overshoots' association with length of stay (LOS), bleeding, and mortality.

**Results** Of the 12,107 older inpatients on chronic warfarin, most were 75 years or older (75.7%), female (51.2%), and white (70.0%). While 1,333 (11.0%) of patients had overshoots during the admission, 449 (33.7%) of these reached overshoots after 48 hours. When stratified by overshoots versus no overshoots, LOS more than doubled (15.6 vs. 6.8 days) and the bleed rate was significantly higher (27.4 vs. 8.3%) in the overshoot group. While overall mortality was small (0.4%), the overshoot group's mortality was significantly higher (3.12 vs. 0.28%). Black race and weight were *protective against* overshoots; history of heart failure and antibiotic/amiodarone exposure were *predictive of* overshoots.

**Conclusion** This is the largest study examining warfarin quality metrics for hospitalized adults, specifically older inpatients. Our model may serve as the basis for identifying high-risk warfarin patients to target interventions to reduce adverse drug events.

## Introduction

The Agency for Healthcare Research and Quality (AHRQ) labels anticoagulants such as warfarin a high-risk drug. Prior studies have shown that the majority of warfarin-associated bleeds result in serious outcomes, with fatal outcomes reported in up to 10%. 1 Warfarin in particular accounts for the largest number of serious adverse event reports in the Food and Drug Administration's (FDA's) Adverse Event Reporting System for the 1990 and 2000 decades, especially in older patients. 1 In a Medicare-specific population, 8.8% of adverse drug events during hospitalizations were attributed to warfarin. 2 In teaching hospitals, one-third of preventable adverse drug events were related to warfarin. 3 These warfarin-associated adverse drug events have a significant economic burden as well; a review of medical and pharmacy claims for patients with atrial fibrillation on warfarin found that annual all-cause health care costs in patients with intracranial or gastrointestinal bleeds amount to $41,903 per patient and $40,586 per patient, respectively, compared with $24,129 per patient on warfarin without bleeding. 4 Given that warfarin-associated adverse drug events are dangerous, common, and costly, the Department of Health and Human Services' National Action Plan for Adverse Drug Event Prevention has identified the safe use of anticoagulation as a national priority. 5 The Joint Commission's 2017 Hospital National Patient Safety Goals specifically recommend in Aim NPSG.03.05.01 to “Take extra care with patients who take medicines to thin their blood.” 6 Achieving anticoagulant safety involves minimizing avoidable adverse drug events, reducing variability in provider care, improving system efficiency, and supporting documentation. 7

Multiple tools exist for warfarin management in the outpatient setting, 8 9 10 11 and dosing algorithms improve time in therapeutic range. 12 Such outpatient protocols include patient factors that may affect sensitivity to warfarin initiation, and subsequently dose adjusting by weekly percentages. 13 14 They are not practical for inpatient use, as they do not account for the nuances of inpatient care, such as frequent use of antibiotics or declining kidney function, and the need to adjust doses on a more frequent basis than every week. Unfortunately, there is a dearth of guidance with regard to inpatient warfarin management. 15 There are very few studies that have explored warfarin management for hospitalized patients; the focus of these studies was on warfarin initiation 16 or these studies did not account for clinical factors affecting maintenance dose. 15 Many studies have supported pharmacy-driven inpatient warfarin management as a method for reducing warfarin-associated adverse drug events. 15 17 18 While this may be effective, it can be impractical, depending on the setting and available resources for dedicating pharmacists to inpatient warfarin management.

We now seek to bridge this gap in warfarin management specifically in the inpatient setting. This study aims to (1) describe a large health system's warfarin quality metrics in older inpatients, defined by the international normalized ratio (INR) control, (2) explore the association between inpatient INR overshoots and clinical outcomes, and (3) identify intrinsic and extrinsic patient factors associated with INR overshoots. We hypothesize that poor warfarin control is common in the inpatient setting and is associated with poor clinical outcomes.

## Methods

We conducted a retrospective chart review at a large health system operating in the New York metropolitan area, encompassing seven hospitals (three tertiary and four community hospitals). Data were extracted from electronic health records of patients 65 years and older who were admitted and treated with chronic warfarin between January 1, 2014, and June 30, 2016. For this study, we defined chronic warfarin as documentation of warfarin use as a home medication prior to admission (i.e., admission medication reconciliation). Our local institutional review board approved the study (IRB #16–642).

We defined INR overshoots as supratherapeutic INRs of greater than or equal to 5; it has previously been shown that the incidence of adverse events, specifically bleeding events, rises steeply with these INR values. 19 To identify the quality of inpatient warfarin as a result of dosing during the acute hospitalization (rather than doses taken prior to admission), we limited our analysis to INRs after the initial 48 hours of the hospital stay. By hospital policy, warfarin dosing required checking daily INRs. To confirm that this policy was followed, we calculated the percentage of INR days as the number of days with INR values available per length of stay (LOS) for the groups with and without INR overshoots.

Data points collected included all inpatient INR values, patient-related variables (age, height, weight, sex, race, marital status, smoking history), and presence of comorbid conditions (myocardial infarction [MI], congestive heart failure [CHF], peripheral vascular disease [PVD], cerebrovascular disease [CVD], dementia, chronic obstructive pulmonary disease [COPD], connective tissue disease, peptic ulcer disease [PUD], diabetes mellitus (DM), moderate or severe chronic kidney disease [CKD], hemiplegia/paraplegia, malignancies, HIV, and liver disease). Additional variables included medications administered during hospitalization (i.e., antibiotics, amiodarone, and statins) and organizational factors (i.e., tertiary vs. community hospital).

Outcomes included hospital LOS, mortality, and clinically significant bleeding. To capture clinically relevant bleeding, patients needed to meet at least two of the following three criteria: (1) an ICD9 code for bleeding (as a hospital diagnosis), (2) RBCs transfused during admission, and/or (3) receipt of a reversal agent during the admission (including any vitamin K, fresh frozen plasma, or prothrombin complex concentrates). The ICD9 bleeding codes used for analysis were derived from members of the New York State Anticoagulation Coalition and from Leonard et al (2008) and are listed in Appendix A . 20 Appendix B clarifies the number of patients who met two or three criteria for bleeding.

**Appendix A:** ICD9 codes bleeding

ICD 9 Code	Definition
2463	Hemorrhage and infarction of thyroid
2554	Corticoadrenal insufficiency
2851	Acute posthemorrhagic anemia
2865	Hemorrhagic disorder due to circulating anticoagulants
2867	Acquired coagulation factor deficiency
2869	Other and unspecified coagulation defect
3361	Vascular myelopathies
36281	Retinal hemorrhage
3636	Choroidal hemorrhage and rupture
36441	Hyphema of iris and ciliary body
3688	Other specified visual disturbances
37272	Conjunctival hemorrhage
37481	Hemorrhage of eyelid
37632	Orbital hemorrhage
37742	Hemorrhage in optic nerve sheaths
37923	Vitreous hemorrhage
38869	Other otorrhea
4230	Hemopericardium
430	Subarachnoid hemorrhage
431	Intracerebral hemorrhage
432	Other and unspecified intracerebral hemorrhage
436	Ill-defined cerebrovascular disease
458	Hypotension
4590	Hemorrhage unspecified
5238	Other specified periodontal diseases
4560	Gastrointestinal hemorrhage of some sort
45520	Gastrointestinal hemorrhage of some sort
45550	Gastrointestinal hemorrhage of some sort
45580	Gastrointestinal hemorrhage of some sort
45620	Gastrointestinal hemorrhage of some sort
53021	Gastrointestinal hemorrhage of some sort
5310	Gastrointestinal hemorrhage of some sort
5312	Gastrointestinal hemorrhage of some sort
5314	Gastrointestinal hemorrhage of some sort
53140	Gastrointestinal hemorrhage of some sort
5316	Gastrointestinal hemorrhage of some sort
532	Gastrointestinal hemorrhage of some sort
5330	Gastrointestinal hemorrhage of some sort
5332	Gastrointestinal hemorrhage of some sort
5334	Gastrointestinal hemorrhage of some sort
5336	Gastrointestinal hemorrhage of some sort
5340	Gastrointestinal hemorrhage of some sort
5342	Gastrointestinal hemorrhage of some sort
5344	Gastrointestinal hemorrhage of some sort
5346	Gastrointestinal hemorrhage of some sort
53511	Gastrointestinal hemorrhage of some sort
53521	Gastrointestinal hemorrhage of some sort
53531	Gastrointestinal hemorrhage of some sort
53541	Gastrointestinal hemorrhage of some sort
53551	Gastrointestinal hemorrhage of some sort
53561	Gastrointestinal hemorrhage of some sort
56202	Gastrointestinal hemorrhage of some sort
56203	Gastrointestinal hemorrhage of some sort
56212	Gastrointestinal hemorrhage of some sort
56213	Gastrointestinal hemorrhage of some sort
56881	Gastrointestinal hemorrhage of some sort
56935	Gastrointestinal hemorrhage of some sort
5789	Gastrointestinal hemorrhage of some sort
5780	Gastrointestinal hemorrhage of some sort
56985	Gastrointestinal hemorrhage of some sort
5351	Atrophic gastritis
5368	Dyspepsia and other specified disorders of function of stomach
53783	Angiodysplasia of stomach and duodenum with hemorrhage
5582	Toxic gastroenteritis and colitis
5738	Other specified disorders of liver
5967	Hemorrhage into bladder wall
5968	Other specified disorders of bladder
5997	Hematuria
59989	Other specified disorders of the urinary tract
6021	Congestion or hemorrhage of prostate
6201	Corpus luteum cyst or hematoma
6228	Other specified noninflammatory disorders of cervix
6238	Other specified noninflammatory disorders of vagina
6262	Excessive or frequent menstruation
6268	Other disorders of menstruation and other abnormal bleeding from female
6269	Unspecified disorders of menstruation and other abnormal bleeding from female
719	Hemarthrosis
7802	Syncope and collapse
7804	Dizziness and giddiness
7807	Malaise and fatigue
78079	Other malaise and fatigue
7827	Spontaneous ecchymoses
7847	Epistaxis
7848	Hemorrhage from throat
7855	Shock without mention of trauma
7863	Hemoptysis
7870	Nausea and vomiting
78799	Other symptoms involving digestive system
7890	Abdominal pain
7899	Other symptoms involving abdomen and pelvis
7992	Nervousness
800–91999	Injury from fall or other causes
925–95999	Other injuries
E8582	Accidental poisoning by agents primarily affecting blood constituents
E880-E88899	Accidental falls
920	Contusion of face scalp and neck except eye
921	Contusion of eye and adnexa
922	Contusion of trunk
923	Contusion of upper limb
924	Contusion of lower limb and other unspecified sites
E9342	Anticoagulants causing adverse effects
E9343	Vitamin K phytonadione causing adverse effects in therapeutic use
E9504	Suicide and self-inflicted poisoning by other specified drugs
E9620	Assault by drugs and medicinal substances
9642	Poisoning by anticoagulants
9643	Poisoning by vitamin K
9645	Poisoning by anticoagulant antagonists
E9804	Poisoning by other specified drugs
9952	Unspecified adverse effect of drugs or medicinal substances
9981	Hemorrhage or hematoma or seroma
5781	Blood in stool
79092	Abnormal coagulation profile

Notes: These codes were derived from members of the New York State Anticoagulation Coalition and primarily from Leonard et al (2008). 20

**Appendix B:** 

Bleeding count	Frequency	Percent	Cumulative frequency	Cumulative percent
0	11,646	66.57	11,646	66.57
1	4,225	24.15	15,871	90.72
2	1,423	8.13	17,294	98.86
3	200	1.14	17,494	100.00

Logistic regression modeling was used to determine the risk factors for INR overshoots. Additional multivariate analysis was employed to associate INR overshoots with LOS, bleeding, and mortality. Variability across the health system was evaluated with INR overshoots by type of hospital, tertiary care versus community facility. Additional analysis of the impact of patient weight (kg) on INR overshoots was done through chi square testing at 10-kg intervals to establish safety thresholds.

## Results

There were 17,494 unique admissions across seven acute care facilities for patients 65 years and over on warfarin. Of these, 12,107 were on chronic warfarin with INR data available and 5,387 were initiated on warfarin during the hospitalization (not included in our target population). Of those on chronic warfarin, 1,020 (8.4%) discontinued warfarin on discharge from the hospital. Patients with INR overshoots were more likely to be discharged without warfarin than those without overshoots (24.3 vs. 7.8%, *p* < 0.0001). Table 1 describes the characteristics of patients on chronic warfarin during their acute hospitalization. The majority of patients (75.7%) were older than 75 years, female (51.2%), and white (70%). One-third had a history of smoking, with the most common comorbid conditions being CHF (46%), DM without chronic complications (31.4%), CVD (24%), COPD (22.9%), moderate/severe CKD (22.2%), and malignancy (22.1%).

**Table 1:** Patient characteristics: 12,107 chronic warfarin inpatients

Characteristics	Total *N* (%)	No overshoots *n* (%)	Overshoots *n* (%)
Age ≥75	9,172 (75.7)	8,834 (75.8)	338 (75.3)
Female	6,203 (51.2)	5,943 (51.0)	260 (57.9)
Race			
White	8,472 (70.0)	8,423 (72.2)	322 (71.7)
Black	1,593 (13.1)	1,570 (13.5)	44 (9.8)
Other	880 (7.3)	1,219 (10.5)	61 (13.6)
Hispanic	704 (5.8)	676 (5.8)	28 (6.2)
Asian	461 (3.8)	449 (3.9)	22 (4.9)
Marital status			
Married	5,496 (45.4)	5,296 (45.4)	200 (44.5)
Widowed	4,144 (34.2)	3,993 (34.4)	151 (33.6)
Single	1,382 (11.4)	1,333 (11.4)	49 (10.9)
Divorced	614 (5.1)	588 (5.0)	26 (5.8)
Other	409 (3.4)	390 (3.3)	19 (4.2)
Separated	65 (0.5)	61 (0.5)	4 (0.9)
Smoker (present/former)	4,035 (33.3)	3,901 (33.5)	134 (29.8)
Comorbid conditions			
CHF	5,569 (46.0)	5,377 (46.1)	192 (42.8)
DM without chronic complication	3801 (31.4)	3,668 (31.5)	133 (29.6)
CVD	2,906 (24.0)	2,786 (23.9)	120 (26.7)
COPD	2,774 (22.9)	2,658 (22.8)	116 (25.8)
Moderate/Severe CKD	2,684 (22.2)	2,571 (22.1)	113 (25.2)
Malignancy	2,675 (22.1)	2,576 (22.1)	99 (22.1)
MI	1,656 (13.7)	1,594 (13.7)	62 (13.8)
PVD	1,609 (13.3)	1,542 (13.2)	67 (14.9)
DM with chronic complication	677 (5.6)	647 (5.6)	30 (6.7)
Connective tissue disease	575 (4.8)	551 (4.7)	24 (5.4)
PUD	458 (3.8)	441 (3.8)	17 (3.8)
Liver disease (moderate/severe)	369 (3.1)	356 (3.1)	13 (2.9)
Hemiplegia/Paraplegia	260 (2.2)	245 (2.1)	15 (3.3)
Metastatic solid tumor	255 (2.1)	245 (2.1)	10 (2.2)
Dementia	135 (1.1)	132 (1.1)	3 (0.7)
HIV	9 (0.1)	9 (0.1)	0 (0)
Medications			
Statins	7,514 (62.1)	7,256 (62.2)	258 (57.5)
Antiplatelets	5,091 (42.0)	4,910 (42.1)	181 (40.3)
Antibiotics	1941 (16.0)	1,837 (15.8)	104 (23.2)
Amiodarone	968 (8.0)	919 (7.9)	49 (10.9)
ICU admission	1,974 (16.3)	1,841 (15.8)	133 (29.6)
First INR	2.6 ± 1.6	2.6 ± 1.6	3.6 ± 2.6

Abbreviations: CHF, chronic heart failure; CKD, chronic kidney disease; COPD, chronic obstructive pulmonary disease; CVD, cerebrovascular disease; DM, diabetes mellitus; ICU, intensive care unit; INR, international normalized ratio; MI, myocardial infraction; PUD, peptic ulcer disease; PVD, peripheral vascular disease.

The percentage of INR days was 96% of the total LOS for both the groups with and without INR overshoots. Table 2 presents supratherapeutic INR rates. Of the 12,107 patients, 5,829 (48.1%) became supratherapeutic with an INR greater than 3 during the admission, and 54.2% of these episodes occurred after the initial 48 hours of hospitalization. While 1,333 (11.0%) of chronic warfarin patients reached an INR greater than or equal to 5 at some point during the admission, 449 (33.7%) of these reached this maximum INR after the initial 48 hours of the hospital stay. Patients with INR overshoots remained over an INR of 5.0 for a mean of 1.9 days (SD: 1.2, range: 1–11 days).

**Table 2:** Supratherapeutic INRs

INR values	No. of patients	%
INR > 3	5,829/12,107	48.1
INR > 3 after 48 h	3,157/9,873	32.0
INR ≥ 5	1,333/12,107	11.0
INR ≥ 5 after 48 h	449/9,873	4.6

Abbreviation: INR, international normalized ratio.

Table 3 presents outcomes stratified by INR category after the initial 48 hours. When stratified by category (INR overshoots: INR ≥ 5 after initial 48 hours vs. no INR overshoots: INR < 5 after initial 48 hours), LOS more than doubled in the group with INR overshoots (6.8 vs. 15.6 days, <0.0001). Overall, the clinically significant bleed rate by our definition was 9%. The group with INR overshoots had a significantly higher bleed rate, compared with the group without INR overshoots (27.4 vs. 8.3%, adjusted odds ratio [OR]: 6.2, *p* < 0.0001). While the overall mortality rate for the chronic warfarin group was small (0.4%), there was a significantly higher mortality rate (3.12 vs. 0.28%, adjusted OR: 8.6, *p* < 0.0001) in the group with INR overshoots. There was no significant difference in either the 30- or 90-day readmission rates between groups.

**Table 3:** Outcomes stratified by INR overshoots

	Overshoots	No overshoots	Adjusted OR	*p* -Value
Length of stay (d)	15.60	6.81	n/a	<0.0001
Mortality	3.12%	0.28%	8.6	<0.0001
Readmissions				
30 d	18.35%	20.71%	n/a	0.1856
90 d	33.28%	31.63%	n/a	0.4647
Bleeding	27.39%	8.27%	6.2	<0.0001

Abbreviation: INR, international normalized ratio.

In evaluating variability in the quality of warfarin management across the seven facilities, the rates of INRs over 5 after the first 48 hours ranged from 3.0 to 5.9%. While there was no significant difference between hospital types (community vs, tertiary) with regard to INR overshoots, admission to a tertiary hospital was found to be protective against bleeding (OR: 0.862, *p* < 0.0016).

Table 4 presents demographic, clinical, and organizational variables used in the prediction model for INR overshoots after the initial 48 hours of hospitalization. Using logistic regression, black race and weight were found to be *protective against* INR overshoots; conversely, history of CHF and antibiotic or amiodarone exposure was *predictive of* INR overshoots. Moderate or severe CKD trended toward predicting INR overshoots, but did not reach statistical significance ( *p* < 0.068). When adding the variable for INR overshoots to the logistic regression model, we found that (in addition to controlling for age, gender, race, smoking status, ICU stay, heart failure, COPD, DM, CKD, malignancy, liver disease, weight, and antibiotic and amiodarone exposure) an INR greater than or equal to 5 was independently predictive of a longer LOS ( *p* < 0.0001), higher bleed rate ( *p* < 0.0001), and higher mortality ( *p* < 0.0001).

**Table 4:** Prediction model for INR overshoots: analysis of maximum likelihood estimates

Parameter	Estimate	*p* -Value
Intercept	−1.00	0.35
Tertiary hospital	−0.01	0.93
Age: years	−0.01	0.14
Gender: male	−0.22	0.10
Race		
Asian	−0.16	0.55
White	−0.27	0.11
Black	−0.59	0.01
Ethnicity: Hispanic	−0.38	0.12
Marital status		
Widowed	−0.27	0.32
Divorced	−0.03	0.92
Married	−0.12	0.64
Separated	0.17	0.79
Single	−0.17	0.56
Smoker	−0.10	0.37
Comorbid conditions		
MI	−0.03	0.82
CHF	−0.22	0.04
PVD	0.10	0.52
CVD	0.14	0.23
Dementia	−1.00	0.16
COPD	0.19	0.11
Connective tissue disease	0.02	0.94
PUD	−0.09	0.74
DM without chronic complication	−0.04	0.72
DM with chronic complication	0.16	0.46
Moderate/Severe CKD	0.23	0.07
Hemiplegia/Paraplegia	0.39	0.19
Malignancy	−0.03	0.79
Metastatic solid tumor	0.03	0.93
Moderate/Severe liver disease	−0.17	0.57
Height	0.00	0.91
Weight	−0.01	<0.01
Medications		
Statins	−0.19	0.07
Antibiotics	0.43	0.00
Amiodarone	0.38	0.02

Abbreviations: CHF, chronic heart failure; CKD, chronic kidney disease; COPD, chronic obstructive pulmonary disease; CVD, cerebrovascular disease; DM, diabetes mellitus; ICU, intensive care unit; INR, international normalized ratio; MI, myocardial infraction; PUD, peptic ulcer disease; PVD, peripheral vascular disease.

Chi-square testing at 10-kg intervals for body weight found significant thresholds at both 50 and 90 kg: 7.7% of patients less than or equal to 50 kg experienced INR overshoots compared with 3.5% of those over 50 kg ( *p* < 0.0001); 4.1% of patients less than or equal to 90 kg experienced INR overshoots compared with 2.5% of patients over 90 kg ( *p* < 0.0001). At weights over 120 kg, there is no significant difference in incidence of INR overshoots between weight classes. Table 5 illustrates an overall downward trend of INR overshoots as weight classes increase, with 7.69% of those 50 kg and under experiencing overshoots, 3.78% of those between 50 and 90 kg, 2.35% between 90 and 120 kg, and 2.98% of those over 120 kg.

**Table 5:** INR overshoots by weight classes

Weight (kg)	No overshoots *n* (%)	Overshoots *n* (%)	Chi-square
≤50	708 (92.31)	59 (7.69)	<0.001
50.01–90	8,116 (96.22)	319 (3.78)	<0.001
90.01–120	2,283 (97.65)	55 (2.35)	<0.001
>120	489 (97.02)	15 (2.98)	<0.001

Abbreviation: INR, international normalized ratio.

## Discussion

This is the largest study to date examining warfarin quality metrics for older adults in the inpatient setting. More specifically, we sought to describe chronic warfarin quality metrics across multiple inpatient facilities, explore the association between inpatient INR overshoots and clinical outcomes, and identify intrinsic patient-related factors and extrinsic factors associated with INR overshoots. Warfarin has previously been described as a high-risk medication, and poor control defined by INR has been associated with negative outcomes; our research is novel in that it specifically investigates the clinical outcomes of INR overshoots in a large hospital-based health system and focuses on risk factors for such overshoots.

Approximately half of the patients on chronic warfarin reached supratherapeutic levels of INR greater than 3 during the admission, and approximately one-half of these occurred after the initial 48 hours of hospitalization. This time frame was used to focus on effects of inpatient, rather than outpatient, events including provider dosing and clinical conditions. Roughly 11% of patients reached supratherapeutic INRs at the clinically important threshold of INR ≥ 5.0, and one-third of these reached this level after the initial 48 hours, with inpatient dosing again likely responsible for these INR overshoots. While the overall bleed rate of 9% is comparable to rates reported in Medicare patients on anticoagulation, 21 the group with INR overshoots had significantly increased LOS, bleeding, and mortality. Given INR overshoots' association with these adverse events, they may serve as potential surrogate markers for identifying such negative outcomes that health systems seek to avoid. By hospital policy, INRs are checked daily for patients presently treated with warfarin. Despite close monitoring with the rate of INR days being 96% in both the overshoot and no overshoot group, there was still poor control in the overshoot group suggesting that frequency of INR checking did not contribute to differences between adverse outcomes between the groups. Future studies should evaluate interventions to improve the safety of inpatient warfarin dosing and may use INR overshoots in addition to clinical outcomes to evaluate the effectiveness of such interventions.

We found that for older patients on chronic warfarin therapy during acute hospitalization, low weight, exposure to antibiotics/amiodarone, and heart failure were independently predictive of INR overshoots, while black race was protective. In additional weight analysis, the largest thresholds for correlation with INR overshoots were found at 50 and 90 kg. At weights above 120 kg, there were no longer associations with INR overshoots. While most of these factors have been identified as markers, or sensitivity classes for outpatient initiation algorithms, this is the first study to evaluate them in an older inpatient population. As examples, the initiation algorithms presented by the University of North Carolina 9 and the University of Wisconsin 10 both include heart failure, low body weight, antibiotics, and amiodarone as intrinsic and extrinsic factors making patients prone to higher warfarin sensitivity. In Kimmel's pharmacogenetics studies, the clinical dose-revision algorithm that was used as a comparison to pharmacogenetics dosing, black race and body surface area were included as factors increasing the recommended warfarin dose, and amiodarone use as a factor lowering the dose. 22

There were several limitations to our study. This was a retrospective chart review, and predictors of INR overshoots were limited by documentation available from the electronic health record with incomplete information, and difficulty establishing cause and effect. Comorbid conditions were identified through an electronic data pull of ICD9 codes alone, and thus we were unable to differentiate between acute, chronic, and past conditions or to establish temporal associations between such comorbid conditions and INR overshoots. Bleeding events during hospitalization could not be time correlated with INR overshoots, again highlighting the lack of ability to establish cause and effect. We do hope to have improved accuracy of bleeding events by requiring a minimum of two bleed-related orders or coding but were unable to verify this through individual chart analysis. Furthermore, this definition of bleeding may have impacted the finding that admission to a tertiary hospital was associated with a lower risk of bleeding due to differences in transfusion management such as evidence-based, higher thresholds for transfusions. Additionally, while ICU admission was more prevalent in the INR overshoot group, we were unable to assess whether such admissions were due to a concurrent illness causing the overshoot, severe bleeding caused by the overshoot, or poor dosing within the ICU. We also did not assess for scenarios when warfarin was intentionally held (i.e., for procedures) or the need for reversal due to bleeding. To overcome this barrier, our focus for poor control was on supratherapeutic INRs known to be high risk for acute bleeding episodes rather than on subtherapeutic INRs. On the inpatient setting, the risk of subtherapeutic INRs can be mitigated by using heparin-bridging therapies when appropriate. We did not separate surgical and medical patients who may have different risk factors for adverse events. A final limitation was that the use of antibiotics or amiodarone was not correlated in its timing with the administration of warfarin.

## Summary and Conclusion

Our study findings indicated that (1) INR overshoots are prevalent in the inpatient setting in an older population across our health system and associated with poor outcomes and (2) INR overshoots are independently associated with low weight, heart failure, non-black race, and antibiotic or amiodarone exposure. In the outpatient setting, both intrinsic patient-related and extrinsic factors are integrated into warfarin dosing algorithms. We found that similar factors are also associated with inpatient INR metrics. Yet, no such tool exists in the inpatient setting to determine appropriate warfarin dosing, especially for more frequent (than weekly) assessments. Our model may serve as the basis for identifying high-risk patients and developing interventions for inpatient warfarin dosing strategies. Future studies should focus on the impact of the rate of change of the INR (the delta INR) on predicting INR overshoots when combined with clinical factors identified by our prediction model. 17
